# Using a Smart Phone as a Standalone Platform for Detection and Monitoring of Pathological Tremors

**DOI:** 10.3389/fnhum.2012.00357

**Published:** 2013-01-18

**Authors:** Jean-François Daneault, Benoit Carignan, Carl Éric Codère, Abbas F. Sadikot, Christian Duval

**Affiliations:** ^1^Department of Neurology and Neurosurgery, Montreal Neurological Institute and Hospital, McGill UniversityMontreal, QC, Canada; ^2^Centre de Recherche de l’Institut, Universitaire de Gériatrie de MontréalMontréal, QC, Canada; ^3^Département des Sciences Biologiques, Université du Québec à MontréalMontréal, QC, Canada; ^4^Département de Kinanthropologie, Université du Québec à MontréalMontréal, QC, Canada

**Keywords:** tremor, telephone, telemedicine, long-term, movement disorder, Parkinson, essential tremor

## Abstract

**Introduction:** Smart phones are becoming ubiquitous and their computing capabilities are ever increasing. Consequently, more attention is geared toward their potential use in research and medical settings. For instance, their built-in hardware can provide quantitative data for different movements. Therefore, the goal of the current study was to evaluate the capabilities of a standalone smart phone platform to characterize tremor. **Results:** Algorithms for tremor recording and online analysis can be implemented within a smart phone. The smart phone provides reliable time- and frequency-domain tremor characteristics. The smart phone can also provide medically relevant tremor assessments. **Discussion:** Smart phones have the potential to provide researchers and clinicians with quantitative short- and long-term tremor assessments that are currently not easily available. **Methods:** A smart phone application for tremor quantification and online analysis was developed. Then, smart phone results were compared to those obtained simultaneously with a laboratory accelerometer. Finally, results from the smart phone were compared to clinical tremor assessments.

## Introduction

Tremor is the most prevalent movement disorder, and can manifest itself in a myriad of pathologies. One of those disorders is Parkinson’s disease (PD) (Calabrese, 2007). According to the World Health Organization, more than 5.2 million people were affected by PD in 2004 (http://www.who.int/healthinfo/global_burden_disease/GBD_report_2004update_part3.pdf), and this number is expected to increase to 40 million by the year 2020 (Morris, 2000). While PD is associated with other motor symptoms besides tremor, it is expected that the majority of patients with PD will exhibit some form of tremor within the course of their disease (Jankovic, 2008). Essential tremor (ET) is another disease exhibiting tremor. It is the most common movement disorder as it affects close to 4000 per 100,000 people aged over 65 years (Louis et al., 1995). The hallmark motor feature of this pathology is postural and action tremor of the upper limb (Louis et al., 1998). While tremor is a cardinal feature in PD and ET, other conditions can also present with tremor; such as dystonia (Jedynak et al., 1991) and multiple sclerosis (Pittock et al., 2004). Finally, tremor can occur as a result of exposure to environmental agents (Louis et al., 2011) or be a side-effect of some medications (Zesiewicz and Sullivan, 2011).

The treatment and monitoring of tremor still represents a significant challenge for clinicians as tremor is highly variable in its characteristics within a day and over several days. Currently, tremor is assessed during clinic visits, mostly with the use of clinical rating scales (see Goetz et al., 2008 for example). However, clinical observations by medical staff are time consuming and not always representative since the testing environment can be stressful for the patient; which can alter symptomatology. Limited methods are currently available to capture and assess tremor over long periods. Patients can manually complete a diary of the evolution of their tremor over several days; however, they may have a difficult time accurately and objectively evaluating their own symptom severity and the effect of medication. Some quantitative methods were developed, such as an electromagnetic tracking device (O’Suilleabhain and Dewey, 2001), a mechanical linkage device on the fingertip (Matsumoto et al., 1999), lasers (Beuter et al., 1994), electromyography (Askari et al., 2010), wearable sensors (Chen et al., 2011), miniature gyroscopes (Salarian et al., 2007), digitizing tablets (Aly et al., 2007), a tremor pen (Papapetropoulos et al., 2010), and accelerometers (Barroso Junior et al., 2011). However, such devices are not readily available to patients or clinicians, they require technical expertise when manipulating hardware and analyzing results, and there are substantial costs associated with their purchase. We propose that smart phones have the potential to address the aforementioned issues. In the coming years, a majority of the population will own smart phones^1^. In fact, research shows that for 2011 alone, more than 420 million smart phones were sold worldwide and these figures are expected to rise to over 1 billion by 2016 (see text footnote 1).

In a recent editorial in Nature methods, it was proposed that smart phones could be used to gather data in the laboratory (No authors listed, 2010). The next step would then be to develop easy-to-use medical applications that correspond to the needs of clinicians, patients, and researchers. Interestingly, almost all smart phones have an accelerometer which, historically, was the device of choice for tremor assessment (Comby et al., 1992). The potential of smart phones for the evaluation of tremor was recently examined by our group (Daneault et al., 2010; Carignan et al., 2011) and others (Lemoyne et al., 2010; Joundi et al., 2011). However, these attempts presented with serious limits since they were performed on limited sample sizes (Lemoyne et al., 2010; Joundi et al., 2011), lacked clinical evaluation, and did not correlate other tremor measurements to the smart phone. Finally, previous studies have not evaluated the possibility that the smart phone could be used as a standalone device for tremor assessment. As such, these previous studies should be considered as proof of principle rather than a validation of the use of smart phones for tremor recordings in research or clinical settings.

To test the real potential of smart phones to assess, characterize, and monitor abnormal tremors, we developed a protocol in two parts. We first determined whether the device could act as a standalone platform for detection and analysis of several tremor characteristics, and whether values obtained with the smart phone were similar to those obtained with a commonly used laboratory measurement tool; i.e., an accelerometer. Secondly, we sought to compare values obtained by the smart phone against those of a clinical evaluation.

## Results

**Figure 1:**
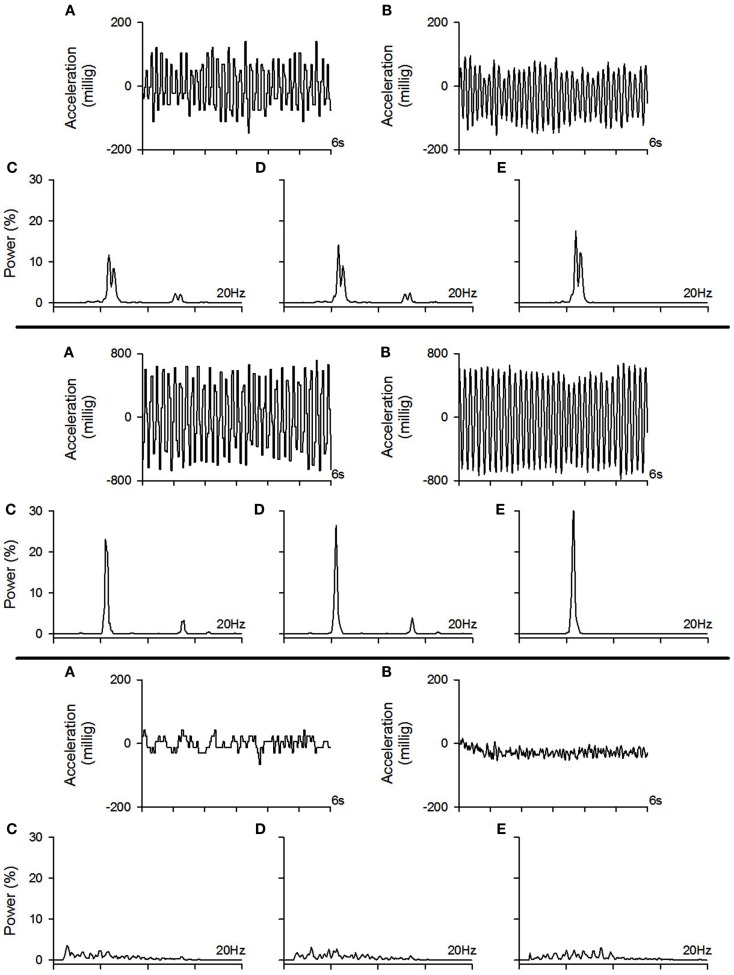
**Example of tremor traces recorded with the smart phone and the accelerometer with their corresponding power spectrum**. *Top pane:* example of a moderate amplitude tremor. *Middle pane:* example of a high amplitude tremor. *Bottom pane:* example of a low amplitude tremor (physiological tremor). **(A)** Example of a tremor trace recorded with the smart phone, **(B)** example of the tremor trace from the same trial as in **(A)** but recorded with the accelerometer, **(C)** power spectrum of the tremor trace recorded with the smart phone which was calculated with the algorithms implemented within the smart phone, **(D)** power spectrum of the tremor trace recorded with the smart phone which was calculated offline using our laboratory software, **(E)** power spectrum of the tremor trace recorded with the accelerometer which was calculated offline using our laboratory software.

In the first part of the study, the objective was to compare a smart phone [Blackberry^®^ Storm™ 9530 (Research In Motion, Ltd., Waterloo, ON, Canada)] to a tremor assessment method normally used in laboratories. In order to do so, tremor was assessed simultaneously with the smart phone and a laboratory accelerometer. One of the authors (Jean-François Daneault) simulated 192 trials of tremor of different amplitudes and frequencies in different conditions (i.e., rest tremor, postural tremor, kinetic tremor, and intention tremor). Analysis of data was then carried out in two steps. In *part 1a*, the objective was to evaluate whether algorithms developed specifically for the smart phone to assess and characterize tremor were as effective as time series analysis software developed for laboratory work. In *part 1b*, the objective was to compare the effectiveness of the smart phone’s accelerometer in replicating results obtained by a laboratory accelerometer. Examples of traces recorded with the smart phone and the accelerometer with their corresponding power spectrum are shown in Figure 1.

In *part 2* of the study, the objective was to evaluate whether tremor amplitude from patients with different pathologies recorded with the smart phone application correlated with tremor amplitude evaluated with a clinical scale. In order to achieve this goal, tremor was assessed simultaneously with the smart phone and a clinical scale in patients presenting with tremor stemming from different pathologies in the conditions stated above. The results from each part of the study are described next.

### Results of part 1a

Both time- and frequency-domain properties of tremor, such as tremor amplitude, tremor regularity, power distribution (percentage of power within the 3–7 Hz frequency band), median power frequency, peak power frequency, power dispersion (frequency band containing 68% of total power centered at the median power frequency), power dispersion centered at peak power frequency, and harmonic index were examined. All these measures are known to help categorize abnormal tremors and provide detailed tremor characteristics (Beuter and Edwards, 1999; Edwards and Beuter, 2000; Duval et al., 2006). To assess the effectiveness of the smart phone’s algorithms, a correlation between values given by the smart phone and those from the post-processing of the time series from the smart phone was performed for each variable of interest (see Table 1). For time-domain characteristics, our results demonstrate that tremor amplitude and tremor regularity presented with a correlation coefficient of 1, regardless of the condition. For frequency-domain characteristics such as the power distribution, median power frequency, power dispersion, and harmonic index, correlation coefficients were always above 0.95. This indicates that the algorithms of the smart phone can accurately replicate the results obtained from the laboratory analysis. As for the peak power frequency, the correlation coefficients were somewhat lower; as they ranged from 0.73 to 0.95, while averaging 0.87. These correlation coefficients are still satisfactory as the 0.73 coefficient can be explained by three outliers that, when removed, allow for the correlation coefficient to rise above 0.90. Then, Bland–Altman and Concordance correlation coefficients (CCC) were computed to assess whether there was a good agreement between both analysis methods. The bias between both analyses methods were mostly below or extremely close to the resolution of the analysis methods, with standard deviations (SD) well within acceptable ranges. CCC were all above 0.95 with several above 0.99 which indicates substantial to almost perfect agreement between methods. The only exception was for the peak frequency where two coefficients were below 0.90. This indicates that the smart phone algorithms could not adequately identify the peak frequency; as such, this variable was not retained for further analysis.

**Table 1:** **Correlation coefficients between the results obtained from the algorithms imbedded within the smart phone and the results obtained from the analysis of the time series from the smart phone by our analysis package using the S-Plus software**.

		*r*	*p*	Bias	SD	CCC
RMS	Rest	1.00	0.00	0.000	0.000	1.00
	Post	1.00	0.00	0.000	0.000	1.00
	Kin	1.00	0.00	0.000	0.000	1.00
	Intention	1.00	0.00	0.000	0.000	1.00
Reg.	Rest	1.00	0.00	0.000	0.001	1.00
	Post	1.00	0.00	0.000	0.001	1.00
	Kin	1.00	0.00	0.000	0.001	1.00
	Intention	1.00	0.00	0.000	0.001	1.00
Pow.Dist	Rest	0.98	0.00	0.723	4.540	0.98
	Post	0.99	0.00	−0.743	2.557	0.99
	Kin	0.99	0.00	−0.246	3.813	0.99
	Intention	0.94	0.00	1.466	7.877	0.94
MPF	Rest	0.99	0.00	0.016	0.194	0.99
	Post	0.99	0.00	−0.031	0.138	0.99
	Kin	0.98	0.00	−0.077	0.242	0.98
	Intention	0.98	0.00	−0.054	0.213	0.98
HI	Rest	0.96	0.00	0.026	0.027	0.96
	Post	0.96	0.00	0.029	0.033	0.96
	Kin	0.97	0.00	0.023	0.019	0.97
	Intention	0.95	0.00	0.023	0.030	0.95
Peak	Rest	0.73	0.00	−0.115	1.157	0.73
	Post	0.86	0.00	−0.096	0.933	0.86
	Kin	0.95	0.00	−0.020	0.540	0.95
	Intention	0.94	0.00	0.093	0.478	0.94
Disp.	Rest	0.99	0.00	0.039	0.348	0.99
	Post	1.00	0.00	0.031	0.216	1.00
	Kin	0.98	0.00	0.189	0.510	0.98
	Intention	0.99	0.00	0.059	0.304	0.99
Disp.Peak	Rest	0.98	0.00	−0.216	0.767	0.98
	Post	0.98	0.00	−0.242	0.692	0.98
	Kin	0.97	0.00	−0.070	0.695	0.97
	Intention	0.99	0.00	−0.125	0.596	0.99

*Correlation coefficients are shown for every task performed. Correlation coefficients were obtained for every variable calculated by the smart phone. RMS, root mean square (tremor amplitude); Reg., tremor regularity; Pow.Dist, power distribution; MPF, median power frequency; Peak, peak power frequency; Disp., power dispersion; Disp. Peak, dispersion centered at peak power frequency; HI, harmonic index; SP, results obtained directly from the smart phone; PP, results obtained by post-processing the time series from the smart phone. Bias between both results and standard deviation (SD) of the difference between results of both methods were computed. Finally, the concordance correlation coefficient (CCC) was also computed between both methods*.

### Results of part 1b

Our results show correlation coefficients above 0.80 for time-domain tremor properties; namely tremor amplitude and tremor displacement regularity (see results in Table 2). While providing fairly similar results to the laboratory assessment tool, the differences observed with the smart phone application could be due to a resolution issue. It seems that the smart phone’s accelerometer is not sensitive enough to properly detect tremor characteristics below a certain amplitude threshold (see bottom pane of Figure 1 for an example a trial near this threshold). Spectral characteristics assessed by the smart phone application seem to vary quite a bit more, as observed correlation coefficients ranged from 0.15 to 0.97, although the majority lie above 0.80. This again could be due to a lack of proper resolution from the smart phone hardware.

**Table 2:** **Correlation coefficients between the results obtained from the algorithms imbedded within the smart phone and the results obtained from the analysis of the time series from the laboratory accelerometer by our analysis package using the S-Plus software**.

		Without threshold		With threshold				
		*r*	*p*	*r*	*p*	Bias	SD	CCC
RMS	Rest	0.99	0.00	0.99	0.00	0.024	0.017	0.99
	Post	0.98	0.00	0.98	0.00	0.022	0.044	0.98
	Kin	0.99	0.00	0.99	0.00	0.071	0.032	0.99
	Intention	0.99	0.00	0.99	0.00	0.066	0.061	0.99
Reg.	Rest	0.92	0.00	0.95	0.00	−0.009	0.053	0.95
	Post	0.81	0.00	0.90	0.00	0.033	0.060	0.90
	Kin	0.88	0.00	0.88	0.00	−0.004	0.046	0.88
	Intention	0.91	0.00	0.98	0.00	0.018	0.039	0.98
Pow.Dist	Rest	0.96	0.00	0.97	0.00	−9.266	8.215	0.97
	Post	0.97	0.00	0.98	0.00	−9.044	9.121	0.98
	Kin	0.97	0.00	0.97	0.00	−7.137	7.208	0.97
	Intention	0.96	0.00	0.92	0.00	−11.259	6.476	0.92
MPF	Rest	0.82	0.00	0.99	0.00	−0.103	0.210	0.99
	Post	0.84	0.00	0.95	0.00	−0.244	0.452	0.95
	Kin	0.59	0.00	0.59	0.00	−0.828	1.268	0.59
	Intention	0.92	0.00	1.00	0.00	−0.160	0.069	1.00
HI	Rest	0.92	0.00	0.92	0.00	0.003	0.010	0.92
	Post	0.77	0.00	0.90	0.00	0.001	0.012	0.90
	Kin	0.81	0.00	0.81	0.00	0.023	0.034	0.81
	Intention	0.89	0.00	0.89	0.00	0.004	0.007	0.89
Disp.	Rest	0.75	0.00	0.55	0.00			
	Post	0.85	0.00	0.81	0.00			
	Kin	0.81	0.00	0.81	0.00			
	Intention	0.96	0.00	0.89	0.00			
Disp.Peak	Rest	0.73	0.00	0.52	0.00			
	Post	0.81	0.00	0.83	0.00			
	Kin	0.79	0.00	0.79	0.00			
	Intention	0.92	0.00	0.87	0.00			

*Correlation coefficients are shown for every task performed. Correlation coefficients were obtained for every variable calculated by the smart phone. RMS, root mean square (tremor amplitude); Reg., tremor regularity; Pow.Dist, power distribution; MPF, median power frequency; Disp., power dispersion; Disp. Peak, dispersion centered at peak power frequency; HI, harmonic index; SP, results obtained directly from the smart phone; Acc, results obtained by post-processing the time series from the accelerometer. Correlations were performed without any threshold to the results as well as after removing all trials having tremor amplitude below 1 mm. Then, for variables showing high correlation coefficients (i.e., RMS, Regularity, Pow.Dist, MPF, and HI), bias between both results and standard deviation (SD) of the difference between results of both methods were computed. Finally, the concordance correlation coefficient (CCC) was also computed between both methods*.

**Figure 2:**
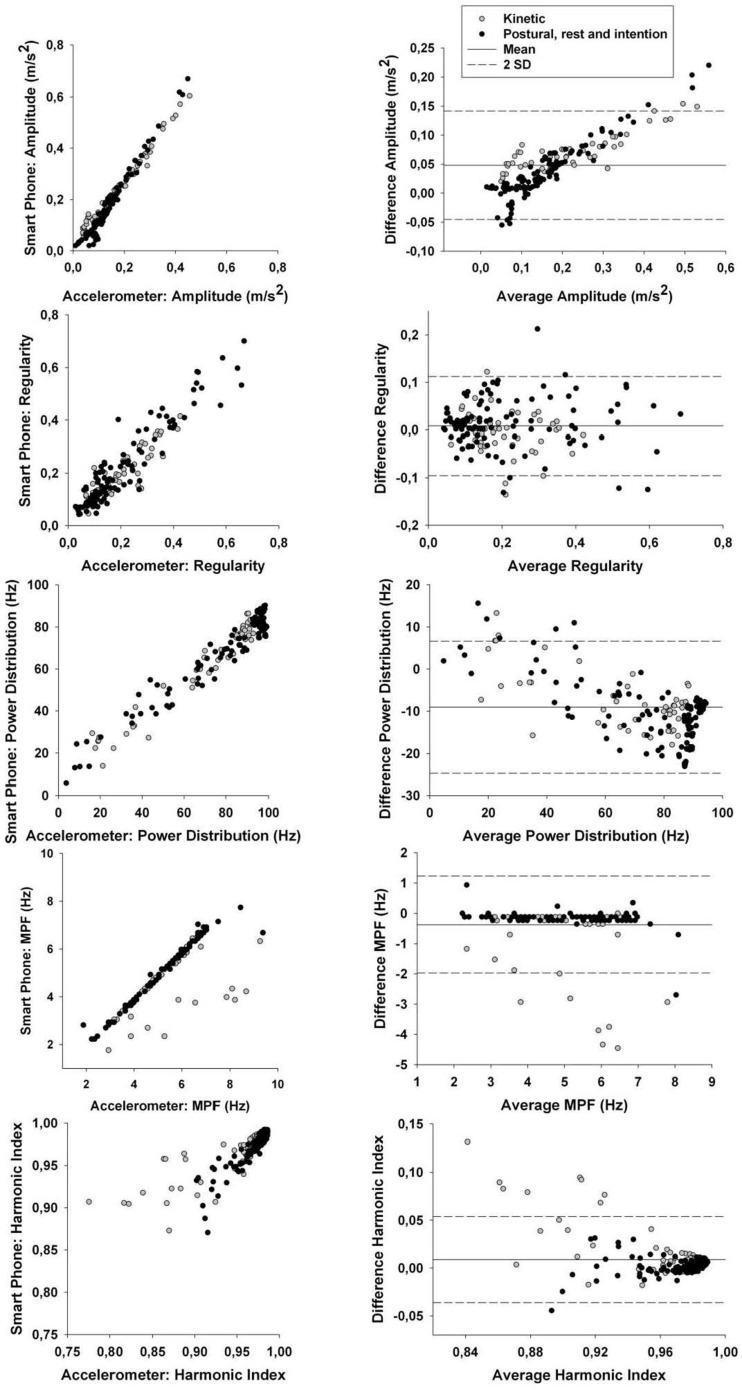
***Left column:* correlation between smart phone data and accelerometer data for tremor amplitude, regularity, power distribution, median power frequency (MPF), and harmonic index (HI)**. Exact correlation coefficients and *p* values are in Table 2. *Right column:* Bland–Altman plots to evaluate the agreement between data from the smart phone and from the accelerometer for tremor amplitude, regularity, power distribution, median power frequency (MPF), and harmonic index (HI). Specific values for the Bland–Altman test (bias, SD) are shown in Table 2.

To verify this, we removed all trials having tremor amplitude below 1 mm from the correlation analysis (see Table 2). This value was chosen as it is at around this amplitude that tremor can become clinically visible. The results demonstrate that correlation coefficients for time-domain tremor properties improved to values above 0.88; with most correlation coefficients being above 0.96. This indicates that limits in the resolution of the hardware within the smart phone caused the lower precision seen in Table 2 before the threshold was applied. The bias and SD between both quantification methods were well within acceptable ranges. CCC were all above 0.95 for RMS and above 0.90 for regularity (except during the kinetic task) which indicates substantial agreement between methods for the RMS and moderate agreement for regularity. As such, the smart phone, using the current algorithms, can be viewed as a valid tool to examine time-domain properties of tremor having amplitude above 1 mm; but not during a kinetic task. This is not a weakness since the goal is to detect abnormal tremor, not physiological tremor. As for frequency-domain tremor properties, correlation coefficients were greatly improved by the implementation of the threshold but not equally for each variable. It seems that power distribution; median power frequency, and harmonic index display the highest correlation coefficients of the spectral variables, whereas power dispersion and power dispersion centered at peak power frequency display less robust correlation coefficients. The low correlation coefficients for both power dispersion calculations may be due to the lower resolution of this tremor characteristic. In fact, as soon as a movement is cyclic and regular, a sharp peak can be observed in the power spectrum, i.e., small power dispersion. Consequently, all simulated tremor over 1 mm had very similar power dispersion in the current data. This lack of variability can explain the poor correlation coefficients observed in this part of the study for these variables. Bias, SD of the difference between both quantification methods and CCC were then computed for spectral characteristics showing high correlations. The bias between both quantification methods were mostly below or extremely close to the resolution of the analysis methods with SD, well within acceptable ranges. CCC were all above 0.95 for the power distribution which indicates substantial agreement between methods. As for the CCC for the MPF and HI, they were above 0.90 with some above 0.95 except during the kinetic task. This indicates that a moderate agreement can be observed between methods except during a kinetic task; where only a poor agreement can be detected between methods. Bland–Altman plots are shown in Figure 2 for tremor characteristics exhibiting high correlations.

As such, the smart phone application could be considered as an adequate measurement tool to provide power distribution, median power frequency, and harmonic index of tremors having amplitude above 1 mm while not performing a kinetic task.

### Results of part 2

To address the objective of this part of the study, we opted to create and validate a clinical rating scale for tremor amplitude (see Table 3). The scale developed allowed for scores between 0 and 5, with each increment being associated with a specific range in tremor amplitude.

**Table 3:** **Table describing the results from the validation of the clinical scale used in *Part* 2 of the study**.

**A. Introduction**			
The purpose of this experiment was to characterize the properties of the clinical scale used to assess tremor amplitude of the hand. The scale incorporated a six level ordinal scale; each associated with a predefined tremor amplitude (0 = no visible tremor; 1 = up to 1.5 cm; 2 = 1.5–3 cm; 3 = 3–4.5 cm; 4 = 4.5–6 cm; and 5 = 6 cm and above)			
**B. Validity**			
		Group 1	Group 2
	
	Rater 1	0,967 (0.000)	0,963 (0.000)
	Rater 2	0,954 (0.000)	0,968 (0.000)
	Rater 3	0,907 (0.000)	0,969 (0.000)
	Rater 4	0,951 (0.000)	0,966 (0.000)
	Rater 5	0,957 (0.000)	0,965 (0.000)
	
Validity of the clinical scale was assessed against a laboratory accelerometer. As such, tremor was assessed simultaneously with both instruments by a group of five raters. This was repeated with a second group of five raters. Pearson correlations were performed between the clinical score and the result obtained from the accelerometer (*p* values are in parentheses beside correlation coefficients)			
**C. Sensitivity**			
Sensitivity of the clinical scale was assessed again against a laboratory accelerometer. Trials were grouped by clinical score and the results obtained from the accelerometer for contiguous clinical scores were compared using repeated *t*-tests with Bonferroni–Holmes adjustments. Asterisks signify a significant difference from the previous box plot. Lines within the box plots represent the median for each group; whiskers represent the tenth and ninetieth percentile and, dots below and above the box plots represent fifth and ninty fifth percentiles, respectively.		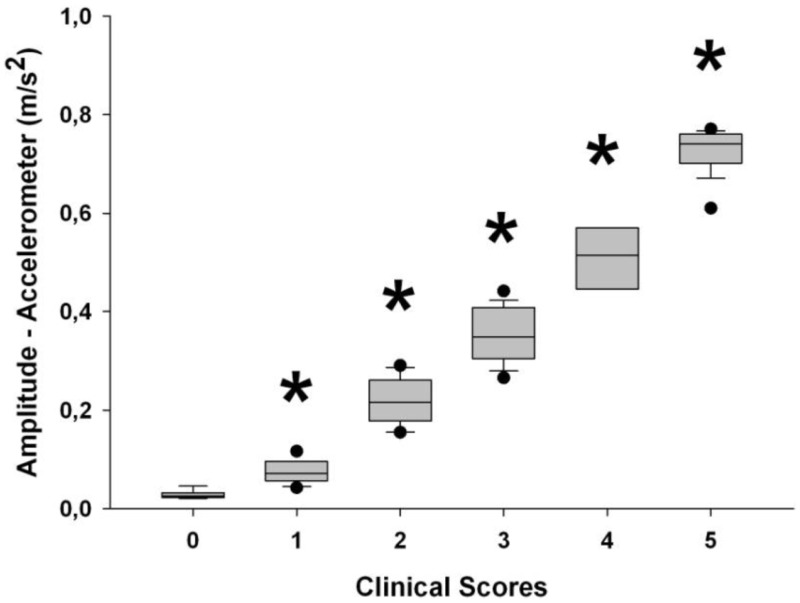	
**D. Reliability**			
		Group 1	Group 2
	
	Intraclass correlation	0,943	0,963
	95% Confidence interval	0,912–0,965	0,945–0,976
	
Inter-rater reliability was assessed using an intraclass correlation from the data of the two groups of five raters with the corresponding 95% confidence interval			
**E. Discussion and conclusion**			
The results presented above demonstrate that the clinical scale used to evaluate hand tremor in the current study is valid, provides good sensitivity and has very good inter-rater reliability. As such, the use of this scale to provide a clinical score of hand tremor is appropriate as per the study parameters			

**Cell A*: description of the clinical scale. *Cell B*: results to demonstrate the validity of the clinical scale. *Cell C*: results to demonstrate the sensitivity of the clinical scale. *Cell D*: results to demonstrate the inter-rater reliability of the scale. *Cell E*: summary of the results and short explanation of their meaning relating to the validity of the clinical scale*.

**Figure 3:**
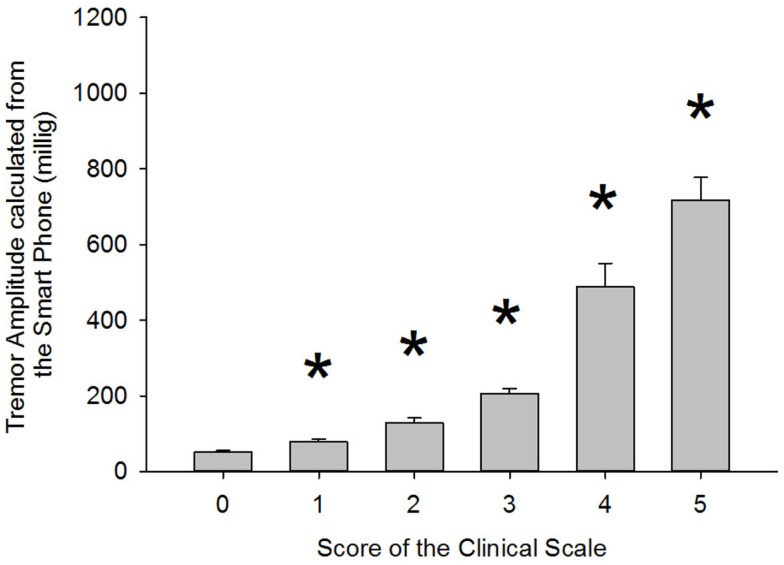
**Comparison of tremor amplitude recorded with the smart phone according to the clinical tremor score each trial was given**. Asterisk (*) indicates a significant difference from the previous group. *p* Values were 0.004, 0.002, 0.001, 0.001, and 0.017 for the paired comparisons 0–1, 1–2, 2–3, 3–4, and 4–5, respectively. The associated power for those tests was 0.788, 0.869, 0.973, 1.000, and 0.638, respectively.

Results presented in Table 4 show that correlation coefficients between recorded tremor and clinical evaluation of tremor were fairly high for the rest, postural and intention tasks with values of 0.76, 0.85, and 0.88, respectively. However, the correlation coefficients were lower than what was seen during the validation of the clinical scale (see Table 3). This can be explained by the fact that pathological tremor can fluctuate even over short periods of time, whereas the simulated tremor used for the validation of the scale was much more constant in amplitude over a given recording. This made the clinical evaluation more difficult. Nonetheless, the correlation coefficients observed with the patients were high enough to expect relevant clinical information from the smart phone application. As for the kinetic task, there was no significant correlation between tremor amplitude calculated by the smart phone and the clinical rating of tremor. This was due to the fact that the very high amplitude of the voluntary movement diminished the influence of tremor. To address this issue, we chose to use the amount of power located within the frequency band associated with tremor (3–7 Hz) as a marker of tremor amplitude. This was done to remove frequencies related to voluntary movements, which lie below 3 Hz. Despite this strategy, the correlation coefficient remained somewhat lower than in other conditions, with a value of 0.7 (see Table 4). We believe that this stems from two factors. First, the clinical evaluation of tremor was more difficult during a voluntary movement. Second, the smart phone application has some difficulty in separating tremor from some of the faster portions of voluntary movements as can be seen from the lower correlation coefficients in that particular task in *Part 1b* of this study (see Table 2). Taken together, these results indicate that the smart phone application can provide relevant clinical information about the amplitude of tremor during static positions, but currently lack specificity during kinetic tasks. Interestingly, when the data are grouped according to clinical rating scores, significant differences can be observed for the tremor recorded with the smart phone application between each clinical score (Figure 3). This demonstrates that the data from the smart phone application can reliably provide a clinical rating of pathological tremor amplitude.

**Table 4:** **Correlation coefficients between the tremor amplitude (RMS) obtained from the algorithms imbedded within the smart phone and the clinical tremor scores**.

		*r* (Pearson)	*p*
Rest	RMS	0.762	<0.000
Postural	RMS	0.851	<0.000
Intention	RMS	0.880	<0.000
Kinetic	RMS	0.086	0.557
	Pow.Dist	0.700	<0.000

*Correlation coefficients are shown for every task performed. Note that for the Kinetic task, since tremor amplitude yielded a poor correlation, the power distribution (Pow.Dist) was chosen to better represent tremor oscillation and was subsequently correlated to the clinical tremor scores. p Values are shown next to their associated *r* values*.

## Discussion

Mobile technology is currently used to transmit or store data obtained from laboratory instruments (Barroso Junior et al., 2011). With the current study, we demonstrate that mobile phones can be used as a standalone platform to assess abnormal motor behaviors such as tremor and as a tool to perform clinical evaluations. More specifically, we demonstrated for the first time that algorithms developed for smart phones can perform as well as time series analysis software used in a laboratory setting to assess and characterize abnormal tremor, as well as differentiate normal from abnormal tremors.

### Validation against laboratory tools

The current study provides a comparison between the use of a smart phone application and laboratory tools for the characterization of tremor. The results demonstrate that the smart phone application can provide similar results to laboratory tools for measurements of time-domain and spectral characteristics albeit with some limitations. While the smart phone always provides valid tremor amplitude values, evaluation of spectral characteristics and regularity of tremor requires an amplitude threshold above 1 mm. This smart phone is therefore not suitable for characterizing the spectral characteristics of normal physiological tremor, but can be used to monitor pathological tremor characteristics. This is expected since accelerometers used in laboratory settings are highly sensitive to low amplitude oscillation but can be deleteriously affected by shocks, which would be impractical in smart phones. Furthermore, our results demonstrate that spectral characteristics of tremor should currently not be assessed during movement. The exact mechanism behind the poorer results obtained during movement is not known, but we hypothesize that it could stem from crosstalk between the three axes of the smart phone accelerometer, which would be absent from the one-axis laboratory accelerometer.

There are numerous ongoing studies examining pathological tremor worldwide. The use of a portable smart phone based measurement tool could therefore provide much needed information on the characteristics of tremor beyond what can currently only be gathered in laboratory setting. Amongst many applications for tremor evaluation, smart phones could provide valid measures of prevalence of ET in different populations, help determine the effect of different medications on tremor frequency and amplitude in PD over the patient’s day, and evaluate the prevalence of tremor as a side-effect of certain medications.

### Validation against a clinical scale

In addition to providing confirmation of the usefulness of smart phones for tremor research, the current study provides for the first time evidence of clinical utility for these devices in tremor disorders. We found a strong relationship between the amplitude of tremor measured by the smart phone application and the amplitude of tremor measured by a clinical rating scale. Furthermore, mean tremor amplitude recorded with the smart phone application for each level of clinical scale rating was significantly different, which indicates that clinicians could obtain a valid profile of tremor severity over time. This demonstrates that the smart phone can provide a reliable measure of tremor of different amplitudes. This is important as it is well known that while not all PD patients exhibit tremor, those that have tremor can exhibit highly variable tremor amplitude and that this amplitude can fluctuate over short periods of time (Duval et al., 1997; Duval and Beuter, 1998; Rahimi et al., 2011). Tremor severity can also fluctuate within a given day and over multiple days which makes long-term monitoring of patients a necessity. This tool could inform the clinician on the true impact of a medication change on the severity and fluctuation of tremor. This is important as a significant part of the costs associated with movements disorders stem from lack of efficiency in adjusting and testing medication efficacy for individual patients. These types of medical tools are then required as the health community tries to move to evidence-based and patient-centered medicine. The health-care system is stretched thin and the development of novel tools to improve efficiency and at the same time improve the quality of life of patients is necessary. While the current study focused on the use of smart phones for the quantification of tremor, using proper algorithms, they could possibly characterize many other forms of involuntary movements.

### Limitations of the current study

The current study examined the feasibility of evaluating tremor using a Blackberry^®^ smart phone. While the data presented in this study clearly demonstrates that several tremor characteristics can be assessed using this device, and that the results provided are clinically meaningful, the algorithms have solely been tested on one Blackberry^®^ model. Indeed, other Blackberry^®^ devices are equipped with the hardware necessary for the proposed applications. We have not yet verified whether other models of Blackberry^®^ smart phones or phones from other manufacturers may indeed be able to characterize the spectral properties of tremor having amplitude below 1 mm. While the feasibility of such applications was briefly demonstrated on the iPhone^®^ (see above), to our knowledge there are currently no such studies on other widely used platforms such as Android™, Java ME, and Windows Phone^®^. The implementation of the method demonstrated in the current study on all major platforms is essential in order to maximize the impact of such a tool. We have no reason to believe that other platforms could not be used for tremor assessment, except if their hardware or software configuration does not allow access to the accelerometer data. It is quite possible that accelerometer chips within smart phones may soon be as precise as laboratory tools and because of their relative affordability, smart phones may eventually replace some laboratory assessment tools.

### Future indications for the use of smart phones in research and medical settings

The current study provides the framework for the use of smart phones for the evaluation of motor behavior. While we presented data on the assessment of tremor, with the proper algorithms, a myriad of other movements, voluntary or involuntary, in health and disease, can be quantified using these devices. The only barrier to the use of smart phones in research settings is creativity and the ability to implement the proper software within the devices to achieve the set goals. The implementation of smart phones in medical settings clearly requires more research since it will directly impact the lives of patients. One foreseeable avenue from the data presented here is the development of applications tailored to the long-term monitoring of patients with tremor. These applications could incorporate testing schedules, data transmission to the physician for remote monitoring, and many other useful specifications. The goal of these applications would be to provide meaningful information to the clinician in order to improve personalized care and reduce burden on the health-care system. Such devices could also greatly improve the efficiency of clinical trials in which tremor monitoring is needed.

## Conclusion

The current study demonstrates for the first time that, with the proper analytical algorithms, smart phones can be used not only for data storage during experiments, but also as a data gathering and analysis tools. Furthermore, we demonstrate that smart phones can provide meaningful and powerful data for clinical evaluations. Smart phones may therefore revolutionize scientific research and greatly improve patient care.

## Methods

### Part 1

The analysis of this part of the study is subdivided into two section; *part 1a* and *part 1b*. The objective of *part 1a* of the study was to evaluate whether our analysis packages normally used to characterize tremor in the laboratory could be translated into a smart phone application yielding corresponding results. The objective of *part 1b* of the study was to evaluate whether the smart phone application yielded similar results as tools commonly used to quantify tremor in the laboratory.

#### Participant

Jean-François Daneault performed the tests. He is free of any neurological disorders that could have influenced movements or understanding of the tasks. Jean-François Daneault is right-handed according to the Edinburg Handedness Inventory. This protocol was approved by the ethics board of the Université du Québec à Montréal.

#### Procedure

##### Apparatus

Tremor was assessed using a Blackberry^®^ Storm™ 9530 (Research In Motion, Ltd., Waterloo, ON, Canada). A choice was made to code the required algorithms for the Blackberry^®^ platform as several were readily available in the laboratory for testing and it was the programming language in which we were the most proficient. Furthermore, the security provided by this platform for storing and transmitting sensitive information that could be medically relevant is currently much greater than on other platforms. Finally, tremor was also quantified using a one-axis accelerometer (TLB333B42, PCB Piezotronics, NY, USA) which was affixed to the back of the smart phone.

##### Design

Tremor was recorded simultaneously with both methods. The accelerometer was fixed to the back of the smart phone using two-sided tape. Smart phone data acquisition rate was set at 60 Hz, and the accelerometer acquisition rate was set at 2048 Hz. For the purpose of the current experiment tremor was always recorded in the same axis (i.e., front-to-back axis of the smart phone). Four tasks were performed by the participant while he was seated: (A) Resting tremor. This task consisted of having the participant sitting with his arm hanging by his side as tremor was being recorded. (B) Postural tremor. This task consisted of having the participant keep his arm and hand outstretched in front of him and parallel to the ground. (C) Intention tremor. This task consisted of having the participant keep his arms and hands in front of him while trying to bring the tips of his fingers as close as possible to each other. (D) Kinetic tremor. This task consisted of starting in the same position as in B and then, bringing the phone to one’s ear and back at a relatively slow velocity. During each task, the participant held the smart phone in his hand. We opted for this approach instead of taping or strapping the smart phone to the participant’s hand because we wanted a more ecological design where they would hold the phone as they would in real life. The participant performed 48 trials of every task while simulating tremors of different amplitude and frequency. No specific instruction on tremor amplitude and frequency was given for each trial; only that after completion, there should be a high variability in tremor amplitude and frequency between trials. Tremor recordings were coded to last 10 s, while only the last 8.5 s of recording were taken into account for analysis in order to minimize the impact of any movement that could have occurred as a result of placing the hand in the required position. A 1-s vibration (∼140 Hz) indicated to the participant the end of each trial. Ten seconds were allotted between trials to minimize fatigue.

### Part 1a

#### Data analysis

The analysis algorithms usually employed in our laboratory, using the S-Plus software (Mathsoft, Seattle, WA, USA), were coded in Java™ in order to run on the Blackberry^®^ operating system. Each trial yielded two files of data on the smart phone. One containing the raw time series of a given trial and another containing the results obtained by the algorithms implemented in the smart phone. The time series was analyzed with both the smart phone algorithms and the algorithms usually employed within the laboratory to obtain tremor characteristics, such as: tremor amplitude, tremor regularity, power distribution, median power frequency, peak power frequency, power dispersion, power dispersion centered at peak power frequency, and harmonic index. Each characteristic and their computation are described below:

##### Tremor amplitude

First, to remove the influence of gravity, the time series was demeaned. Then, a root mean square was applied to the signal.

##### Tremor regularity

First, the original time series was normalized, i.e., the mean of the time series was removed from each point and then, each point was divided by the SD of the time series. After, the signal was divided into epochs of 1 s and the amplitude (root mean square) was calculated for each epoch. Finally, the total SD from the amplitude of all epochs was computed. This yielded a measure of signal amplitude stability over time. A more regular signal was associated with a lower value.

Spectral characteristics of tremor were then evaluated. To do so, a fast-Fourier transform (FFT) was performed on the time series. Codes were written in such a way that the power spectral density function yielded only the power lying between 1 and 20 Hz as these are the prominent frequencies of tremor.

##### Power distribution

Represents the sum of the power within a specific frequency band located between 3 and 7 Hz; divided by the total power. This frequency band harbors the majority of power in most pathological tremors (McAuley et al., 1997).

##### Median power frequency

Represents the frequency where 50% of the power lies below it and the remaining 50% lies above it.

##### Peak power frequency

Represents the frequency where the maximum power was observed.

##### Power dispersion

Represents the width of a frequency band containing 68% of total power; centered at the median power frequency.

##### Power dispersion centered at peak power frequency

Represents the width of a frequency band containing 68% of total power; centered at the peak power frequency.

##### Harmonic index

Represents a ratio considering a rectangle bounded on the sides by the frequency band of interest (0–20 Hz), and vertically from 0 to the height of the highest peak. The harmonic index is the proportion of the area of this rectangle lying above the power spectrum itself.

While the coding of the algorithms mentioned above provide the analytical basis of the application, several lines of codes were also required to implement the graphical user interface, to assign a specific id to each recording, to record files in appropriate folders on the phone, and many more functional issues that are required when using a smart phone application. These will not be discussed in detail here as they can be modified to suit the needs of each study and do not directly impact the data being analyzed.

#### Statistical analysis

In order to identify whether the algorithms written for the smart phone provide the same results as those used in the laboratory, a Pearson’s correlation was performed on the data. The results provided by the smart phone were compared to results obtained by analyzing the raw time series of the smart phone offline using custom-designed algorithms within the S-Plus software (Mathsoft, Seattle, WA, USA). These analyses have been used on several occasions by our group to characterize tremor in healthy and pathological populations (see Duval et al., 2006; Carignan et al., 2010 for example).

Next, Bland–Altman analyses were performed between data from the smart phone and the accelerometer for each variable (bias and SD of the difference between both methods of analysis).

Finally, concordance correlation coefficients (CCC) were computed between data from both methods. These last two analyses were performed to identify whether there was a good agreement (reproducibility) between both tremor analysis methods.

### Part 1b

#### Data analysis

Note that for this part of the study, the data from the accelerometer was compared to the results calculated by the smart phone; not from post-processing of the phone time series. Time series from the accelerometer were recorded on a computer for post-processing.

First, in order to identify whether the data recorded by the phone was congruent with measures obtained with the accelerometer, the time series obtained with the accelerometer were analyzed using the S-Plus software (Mathsoft, Seattle, WA, USA). Data from the accelerometer were down-sampled to 60 Hz using a moving average. The same analysis performed in *Part 1a* of the study was carried out on the accelerometer time series.

#### Statistical analysis

In order to identify whether the results stemming from the algorithms written for the smart phone are significantly correlated with results stemming from the analysis of the time series of a laboratory accelerometer, a Pearson’s correlation was performed. First, a Pearson’s correlation was performed on all trials pooled together. Then, a Pearson’s correlation was performed on trials within each condition.

Then, to assess whether a threshold of tremor amplitude would improve the correlation between instruments, the same correlations were performed only on trials having tremor amplitude above 1 mm.

Next, Bland–Altman analyses were performed between data from the smart phone and the accelerometer for each variable showing high correlation between methods (i.e., RMS, regularity, MPF, distribution, and harmonic index).

Finally, CCC were computed between data from both methods for the same variables showing high correlations. These last two analyses were performed to identify whether there was a good agreement (reproducibility) between both tremor quantification methods.

### Part 2

The objective of this part of the study was to evaluate whether there was a relationship between the results from the smart phone application and a clinical scale to evaluate tremor amplitude.

#### Participants

Sixteen patients were recruited for this experimentation. Twelve patients were diagnosed with idiopathic PD. Three patients were diagnosed with ET and one patient was diagnosed with multiple sclerosis. When patients exhibited clinically visible tremor within one limb, tremor recordings were performed on that limb otherwise, tremor was recorded on their dominant side. The experimental protocol was approved by the institutional ethics board of the Montreal Neurological Hospital and Institute. Fourteen patients were right-handed and two were left-handed.

#### Procedure

Tremor was concurrently evaluated using two methods: the smart phone and a clinical scale performed by Jean-François Daneault. The clinical scale was custom-designed to evaluate tremor amplitude of the upper limb. The rater was asked to assign a value ranging from 0 to 5 to the participant’s tremor. Each value was representative of a specific tremor amplitude [0 = no visible tremor; 1 = up to 1.5 cm (roughly the equivalent of one finger width based on a previous study; Peters et al., 1990); 2 = 1.5–3 cm (more than the width of one finger up to two fingers); 3 = 3–4.5 cm (more than the width of two fingers up to three fingers); 4 = 4.5–6 cm (more than the width of three fingers up to four fingers); and 5 = 6 cm and above (more than the width of four fingers)]. We evaluated the specificity and reliability of this tremor amplitude rating scale prior to clinical testing (see Table 3). The reasons behind the development of this scale rather than using an already available tremor rating scale for PD are that: *(a)* the scale needed to provide a quantitative measure of tremor amplitude, *(b)* the scale needed to provide a high degree of precision (small increments between ordinal values), and *(c)* the scale needed to be linear. The most commonly used clinical scales that assess tremor in PD do not provide all three characteristics. The Unified Parkinson’s Disease Rating Scale (UPDRS) (Fahn et al., 1987) does have two items in the motor section to assess tremor however, it does not provide quantitative measures of amplitude. The Movement Disorders Society revision of the UPDRS (MDS-UPDRS) (Goetz et al., 2008) does provide quantitative measures of tremor amplitude however, the increment in clinical score does not reflect a linear increase in tremor amplitude and it does not provide a measurement precise enough for our purpose. Another scale that is sometimes used is the Fahn, Tolossa, Marin tremor rating scale. Again, this scale does not provide quantitative measures of tremor amplitude. As such, we deemed necessary to develop our own scale.

Patients were asked to perform the four tasks that were evaluated in *Part 1a and 1b* of the current study. Each task was repeated three times and a rest period of 20 s was allotted between trials to minimize fatigue.

#### Data analysis

Tremor amplitude from the smart phone was analyzed using the same method as described in *Part 1a*. However, tremor amplitude for all three axes were computed and compared. The highest tremor amplitude value was retained for comparison with clinical scores.

#### Statistical analysis

In order to identify whether the amplitude results stemming from the smart phone are significantly correlated with results stemming from a clinical rating scale, a Pearson’s correlation was performed within each condition. Then, we evaluated whether the smart phone was able to detect clinical differences. For this, the amplitude of each trial assigned a given clinical score were pooled. Then, the mean tremor amplitude was computed for each clinical rating (0–5). The mean value for each contiguous score was compared using a *t*-test (0–1, 1–2, 2–3, 3–4, and 4–5). Initial threshold for significance was set at *p* > 0.05. To correct for multiple comparisons, a Bonferroni–Holmes adjustment was performed.

## Conflict of Interest Statement

Jean-François Daneault, Benoit Carignan, Carl Éric Codère, and Christian Duval have partial ownership of Medapplets, Ltd.; a company created for the development of medical applications for mobile devices. This company has not commercialized any application for mobile devices as of the date of manuscript submission. No commercial application has emerged from the data presented in this manuscript as of the date of submission.

## Footnotes

^1^Smart Phone Sales Statistics. According to figures for 2010, smart phones accounted for 297 million (19%) of the 1.6 billion mobile phones sold that year (http://www.gartner.com/it/page.jsp?id=1543014). US sales of smart phones are expected to grow from 67 million in 2010 to 95 million in 2011, and become the highest-selling consumer electronic device category (http://www.gartner.com/it/page.jsp?id=1550814). Currently, US smart phone ownership is estimated at just over 37% of mobile subscribers or 87.4 million people as of September 2011 (http://www.comscore.com/Press_Events/Press_Releases/2011/11/comScore_Reports_September_2011_U.S._Mobile_Subscriber_Market_Share). These figures are also representative for France, Germany, Italy, Spain and the UK where the combined smart phone ownership is estimated at 38.9% or 91.4 million people (http://www.comscore.com/Press_Events/Press_Releases/2011/10/Smartphones_and_Tablets_Drive_Nearly_5_Percent_of_Digital_Traffic_in_EU5). Sales predictions estimate that 420 million smart phones will be sold in 2011 such that it will represent 28% of the mobile handset market. This figure is predicted to rise to over 1 billion in 2016 which will represent half of the total cellular phone market (http://imsresearch.com/pressrelease/Global_Smartphones_Sales_Will_Top_420_Million_Devices_in_2011_Taking_28_Percent_of_all_Handsets_According_to_IMS_Research).
